# Cytomegalovirus Retinitis as a Sole Manifestation of HIV Infection

**DOI:** 10.7759/cureus.18642

**Published:** 2021-10-10

**Authors:** Cláudia Ferreira Tátá, Tiago Ramires, Maria Piteira, Rui Matono, Claudiu Guz

**Affiliations:** 1 Internal Medicine Department, Hospital do Espirito Santo de Évora, Évora, PRT

**Keywords:** retinal hemorrhage, opportunistic infections, retinitis, hiv, cytomegalovirus

## Abstract

Retinitis is the most frequent manifestation of Cytomegalovirus (CMV) disease in patients with HIV infection. The virus reaches the retina by hematogenous spread, therefore patients with serum CMV load are at increased risk of developing CMV retinitis. The evolution of retinitis without specific treatment causes irreversible visual loss. Proper treatment is essential for controlling the disease progression, prevention of relapses, and contralateral eye involvement.

This report describes a 56-year-old white male who started a progressive decrease in visual acuity (VA) of the right eye, without pain or inflammatory signs. Initial fundoscopy identified a dispersed preretinal hemorrhage and yellowish exudates. For the hypothesis of CMV retinitis, serology for HIV was requested and the subsequent result was positive. Other opportunistic infections, as well as manifestations of CMV infection in other organs, were ruled out. The patient was discharged on valganciclovir and highly active antiretroviral therapy (HAART) with progressive improvement in retinal changes, but without full recovery from VA due to chronic vitritis and tractional retinal detachment. Slow recovery of lymphocyte populations and sustained decrease in viral load were observed.

CMV retinitis as an initial and sole manifestation of HIV infection is rare and requires screening. The importance of this case lies in its rarity, since CMV retinitis was the only manifestation of CMV infection and the only opportunistic infection in this patient. Early diagnosis and initiation of targeted therapy decrease the morbidity associated with this infection.

## Introduction

Cytomegalovirus (CMV) retinitis is the most common sight-threatening intraocular infection in HIV-infected patients. CMV belongs to the herpesviridae family and it is estimated that about 50-80% of the adult population has been exposed to the virus [1,2]. CMV is ubiquitous in humans but it is usually dormant and asymptomatic among immunocompetent patients [1,2]. The first infection in immunocompetent individuals is usually asymptomatic or similar to a mononucleosis syndrome, inducing a primary and consequent immune response establishment of long-term immunity. However, the virus remains latent in the body. The virus reaches the retina by hematogenous spread, therefore patients with serum CMV load are at increased risk of developing CMV retinitis. Prolonged states of immunosuppression can lead to uncontrolled replication and the onset of serious illness, patients with HIV constitute a risk group. Retinitis is the most frequent manifestation of CMV disease in patients with HIV infection [3]. Prior to the introduction of highly active antiretroviral therapy (HAART), CMV retinitis occurred approximately in 25-40% of AIDS patients [3,4]. The evolution of retinitis without specific treatment causes irreversible visual loss.

## Case presentation

**Figure 1:**
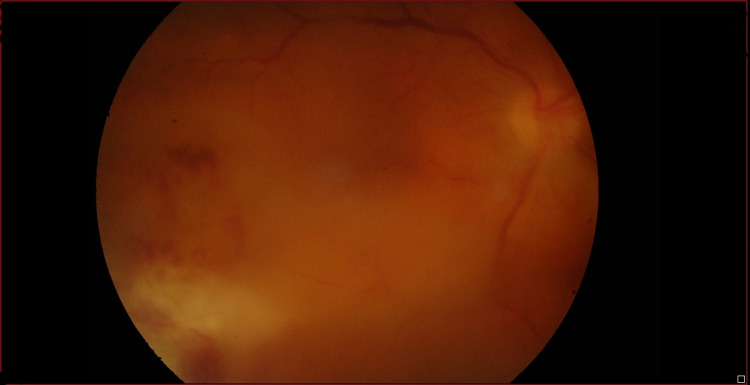
Retinography of the right eye on admission. Hemorrhage and exudates.

**Figure 2:**
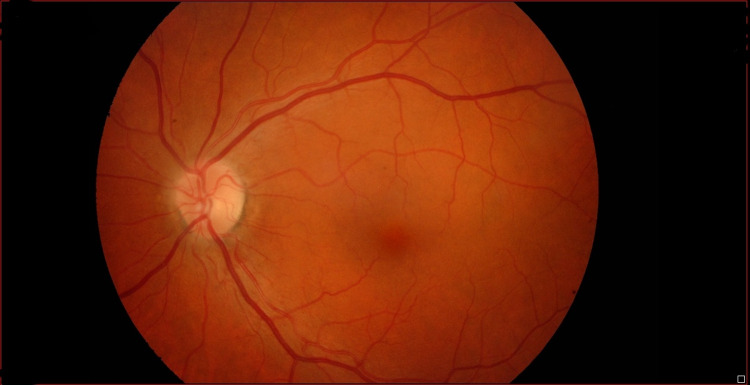
Retinography of the left eye. Not affected.

We present a 56-year-old white male presenting with a history of acute myocardial infarction with high tobacco and alcohol consumption. The patient refers to risky sexual behaviors, but no use of IV drugs. He went to an ophthalmology consultation because of a progressive decrease in visual acuity (VA) of the right eye (RE), in the last few months, without pain or inflammatory signs. Fundoscopy revealed dispersed preretinal hemorrhage and yellowish exudates. CMV retinitis was established as the main diagnostic hypothesis in the context of a possible HIV infection. HIV serology was requested, and a positive result was found. The patient was referred for an Internal Medicine consultation. While waiting for an appointment, he presented a rapidly progressive worsening of his VA and resorted to the Emergency department. Upon observation, he maintained alterations in the retinography of the RE, with worsening of pre-retinal hemorrhage and yellowish exudates, with no other findings (Figure 1). The left eye was not affected (Figure 2).

He was admitted to the Internal Medicine department, assuming the diagnostic hypothesis of CMV retinitis as the first manifestation of HIV infection and starting IV ganciclovir empirically.

An etiological study was performed, showing positive CMV serology for acute infection with positive IgM and IgG and confirmation of HIV type 1 infection by the western blot technique, with a viral load of 13076 copies/mL and decreased CD4+ T lymphocytes (155cel/μL). Confirmed CMV and HIV-1 infection, with criteria of AIDS, in stage C, according to the classification proposed by CDC Atlanta for patients infected with HIV. He started HAART with tenofovir, emtricitabine and efavirenz, and Pneumocystis *jirovecii* pneumonia prophylaxis. Other opportunistic infections, as well as manifestations of CMV infection in other organs, were ruled out. The sexual partner's HIV infection was excluded. The patient was discharged maintaining the therapy mentioned above.

Follow-up consultation revealed good therapeutic adherence, with the slow recovery of lymphocyte populations and a sustained decrease in viral load.

**Figure 3:**
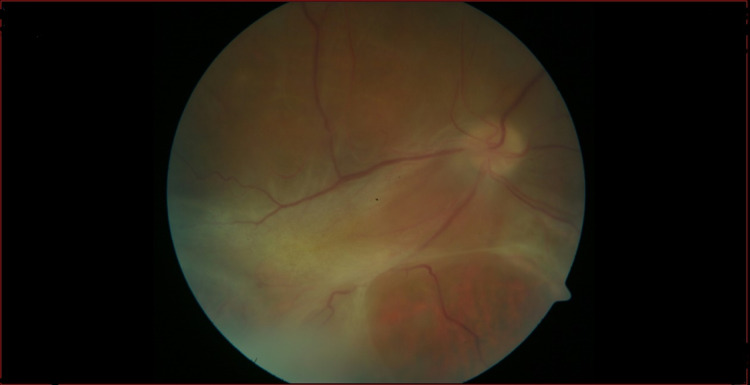
Retinography of the right eye performed three months after the beginning of therapy. Resolution of hemorrhages and exudates. Retinal fibrosis and retinal detachment.

**Figure 4:**
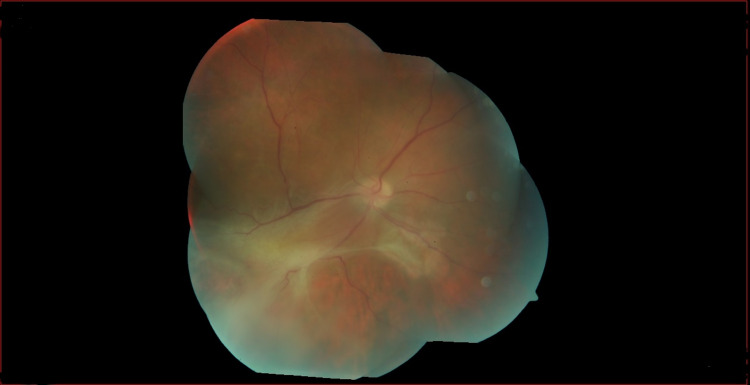
Widefield retinography of the right eye performed three months after the beginning of therapy. Resolution of hemorrhages and exudates. Retinal fibrosis and retinal detachment.

Ophthalmology follow-up one month after treatment showed an improvement in retinal bleeding and exudates. Two months later, the retinography (Figure 3) and the widefield retinography (Figure 4) show a total resolution of bleeding and exudates but no full VA recovery was achieved due to chronic vitritis and tractional retinal detachment. Vitrectomy was not indicated at the moment by the ophthalmology department due to necrosis and tractional detachment.

Three months Internal Medicine follow-up shows clear immunological improvement with T lymphocytes CD4+ 333 cel/µL and viral load 96 copies/mL. Pneumocystis *jirovecii* pneumonia prophylaxis was discontinued, maintaining HAART, valciclovir, and serial ophthalmology follow-ups.

## Discussion

CMV retinitis is presented in patients who have impaired T-cell function usually as a result of HIV infection. It is rare in patients under iatrogenic immunosuppression due to organ or bone marrow transplants [5]. With HAART, the prevalence of CMV retinitis has decreased by about 80 to 90%, currently occurring mainly in cases of advanced immunosuppression, usually in patients with CD4+ T lymphocyte counts below 100 cells/mm3, that are not complying with HAART, present resistance, or that do not respond to this therapy [3]. As such, there is a need for surveillance eye care of patients in order to allow an early diagnosis and adequate treatment. Diagnosis is clinical, based on symptoms and fundus findings. The main symptoms are a decrease in VA (67%), floaters (49%), and flashing lights (16%), also ocular irritation may present [6]. Fundus findings are necrotizing hemorrhagic retinitis, typically retinal microinfarctions, and bleeding along a vascular path. In addition to the classic presentation, the disease may appear with the same lesions without hemorrhage or with retinal detachment. Retinitis usually has a perivascular distribution, it can occur in any part of the retina and have more than one active focus. The lesion progression speed is slow in most cases, a typical expansion rate of 250-350 µm/week, as opposed to other infectious retinitis. Proper treatment is essential for controlling the disease progression, prevention of relapses, and contralateral eye involvement.

HIV infection usually causes AIDS, defined by numerous clinical findings that commonly are multiple at the moment of diagnosis. Nevertheless, AIDS may be diagnosed by the presence of only one secondary infection as shown in our clinical case. A retrospective study refers that 15% of CMV retinitis cases positive for HIV did not have a previous diagnose of AIDS at the moment of consultation [7]. Of the 100 patients studied, in 9% of the cases, CMV retinitis was the only disorder found that meet CDC criteria for AIDS diagnose. Based on these numbers they estimate that only 18% of the patients with AIDS would present with CMV retinitis as the first manifestation of the syndrome [7]. CMV retinitis as an initial and sole manifestation of HIV infection is rare and requires screening. Early diagnosis and initiation of targeted therapy decrease the morbidity associated with this infection.

## Conclusions

The importance of this case lies in its rarity, since CMV retinitis was the only manifestation of CMV infection and the only opportunistic infection in this patient.

We think good and fast interdepartmental communication between ophthalmology and internal medicine must be encouraged in order to achieve a prompt AIDS diagnosis and preserve visual function as long as other complications related to HIV infection are improving.
